# Retinograd-AI: An Open-Source Automated Fundus Autofluorescence Retinal Image Gradability Assessment for Inherited Retinal Diseases

**DOI:** 10.1016/j.xops.2025.100845

**Published:** 2025-06-04

**Authors:** Gunjan Naik, Saoud Al-Khuzaei, Ismail Moghul, Thales A.C. de Guimaraes, Sagnik Sen, Malena Daich Varela, Yichen Liu, Pallavi Bagga, Vincent Rocco, Dun Jack Fu, Mariya Moosajee, Savita Madhusudhan, Andrew R. Webster, Samantha De Silva, Praveen J. Patel, Omar A. Mahroo, Susan M. Downes, Michel Michaelides, Konstantinos Balaskas, Nikolas Pontikos, William Woof A.

**Affiliations:** 1 Faculty of Brain Sciences, UCL Institute of Ophthalmology, London, United Kingdom; 2 Moorfields Eye Hospital NHS Foundation Trust, London, United Kingdom; 3 NIHR Biomedical Research Centre at Moorfields Eye Hospital NHS Foundation Trust and UCL Institute of Ophthalmology, London, UK; 4 Oxford Eye Hospital, John Radcliffe Hospital, Oxford, UK; 5 Nuffield Laboratory of Ophthalmology, Nuffield Department of Clinical Neuroscience, University of Oxford, John Radcliffe Hospital, Oxford, UK; 6 St Paul’s Eye Unit, Liverpool University Hospitals NHS Foundation Trust, Liverpool, UK; 7 Department of Ophthalmology, Faculdade São Leopoldo Mandic, Campinas, São Paulo, Brazil

**Keywords:** AI, Gradability, Fundus Autofluorescence, Inherited retinal diseases, Image quality

## Abstract

**Purpose:**

To develop an automated system for assessing the quality of fundus autofluorescence (FAF) images in patients with inherited retinal diseases (IRDs).

**Design:**

A retrospective study of imaging data.

**Participants:**

Patients with a confirmed molecular diagnosis of IRD who have undergone FAF imaging at Moorfields Eye Hospital.

**Methods:**

A dataset of 2445 FAF images from patients with IRD was marked by 3 expert graders as either gradable (acceptable quality) or ungradable (poor quality), following a strict grading protocol. This dataset was used to train an artificial intelligence (AI) algorithm, Retinograd-AI, which was then applied to predict the gradability label of our entire dataset of 136 631 FAF images.

**Main Outcome Measures:**

Fundus autofluorescence gradability of FAF images as predicted and validated against human assessment.

**Results:**

Retinograd-AI achieves 91% accuracy on our held-out dataset of 133 images with an area under the receiver operator characteristic curve of 0.94, indicating high performance in distinguishing between gradable and ungradable images. Applying Retinograd-AI to our entire dataset, a small but significant positive association of gradability with age was found (ß = 0.002, *P* < 0.001). Excluding X-linked conditions, 77.1% of images were rated as gradable in men and 82.3% in women (odds ratio = 1.43, *P* < 0.001). By genotype, from the 30 most common genetic diagnoses in our dataset, the highest proportion of gradable images was in patients with disease-causing variants in *PRPH2* (93.1%), while the lowest was in *RDH12* (27.1%). Applying Retinograd-AI to filter images improved the accuracy of a gene prediction classifier from 33.8% to 68.9%. Retinograd-AI is open-sourced and available at https://github.com/Eye2Gene/retinograd-ai.

**Conclusions:**

Retinograd-AI is an open-source AI model for automated retinal image quality assessment of FAF images in IRDs. Automated gradability assessment through Retinograd-AI enables large-scale analysis of retinal images and the development of robust analysis pipelines. Quality assessment is essential for the deployment of AI algorithms, such as Eye2Gene, into clinical settings. Due to the diverse nature of IRD pathologies, Retinograd-AI will be extended to other conditions, either in its current form or through transfer learning and fine-tuning.

**Financial Disclosure(s):**

Proprietary or commercial disclosure may be found in the Footnotes and Disclosures at the end of this article.

Inherited retinal diseases (IRDs) are genetically determined disorders of the retina, which collectively represent a leading cause of blindness in children and the working-age population. Inherited retinal diseases encompass a wide range of conditions, with 280 different associated genes identified so far.^1^, ^2^, ^3^ Retinal imaging, using various imaging modalities, allows accurate phenotyping, which is important in the diagnosis and follow-up of IRDs.

Fundus autofluorescence (FAF) imaging is particularly important in this regard since it can yield information relating to the outer retina and retinal pigment epithelium. For instance, an increased autofluorescent signal (hyper-autofluorescence) can result from the accumulation of autofluorescent material, such as lipofuscin, or from the loss of either photoreceptor outer segments or macular luteal pigment, which usually absorbs the incoming short wavelengths.^4^ Similarly, loss of autofluorescence can be associated with the loss of retinal pigment epithelium. Patterns of autofluorescence are associated with certain IRDs, such as the hyperautofluorescent flecks that are usually seen in Stargardt disease.^5^

However, numerous imaging factors can affect imaging quality, such as low signal-to-noise ratio (SNR) during image acquisition resulting in grainy or noisy images; patient movement during image capture, which may make it difficult to acquire macula-centered images; artifacts due to environmental static magnetic waves; interference of external light resulting in bad illumination while scanning; and defocused images due to poor acquisition technique. The quality of imaging data is a critical factor for developing artificial intelligence (AI) models and during the selection of scans for training AI models such as Eye2Gene and AIRDetect.^6^, ^7^, ^8^ Image quality significantly influences model performance and uncertainty metrics in image classification or segmentation. Poor-quality images frequently cause AI model failures, whereas clinicians would disregard these as ungradable or request repeat imaging.

Image gradability refers to if an image is sufficient for a human (or AI) specialist to make an informed decision based on the image. Although gradability is technically distinct from image quality, these aspects are highly correlated, and in the literature, the terms are often used interchangeably.^9^ Manually grading images is laborious and subjective, which highlights the need for automated gradability assessment to filter out poor-quality imaging data. This is crucial for selecting images for training AI models and for using these models to assess biomarkers in clinical trials. These approaches are also necessary for deployment of AI systems in a routine clinical care setting by employing automated grading as a prefiltering step to assess whether repeat imaging is necessary and prevent propagation of decisions based on unreliable data.

Several AI models for retinal image quality assessment of color fundus images have been developed,^10^, ^11^, ^12^, ^13^, ^14^ as well as a few for assessing the quality of spectral-domain OCT images.^15^^,^^16^ However, no models currently exist for other modalities such as FAF, and none have been specifically developed for the gradability of IRDs.

Assessment of gradability of retinal scans from IRD patients poses unique challenges due to IRDs having a range of phenotypes depending on the gene involved. For example, significant areas of decreased autofluorescence can make it hard to discern other features, such as the retinal vasculature. Distinguishing these pathological features from other imaging artifacts is challenging but is crucial for reliable grading and the proper functioning of AI models. Hence, in addition to their application to IRDs, IRD datasets may be a good starting point for developing more general gradability assessment models, as they encompass a wide range of different conditions and pathologies and affect patients across all age ranges.

We present Retinograd-AI, the first retinal image gradability assessment tool for FAF imaging and the first specifically developed for all types of IRDs. Retinograd-AI is a deep neural network–based classifier trained and validated on >2400 FAF images from patients seen at Moorfields Eye Hospital, annotated by 3 expert graders (S.A.K., T.A.C.G., S.S.).

## Methods

This research was approved by the Institutional Review Board and the UK Health Research Authority Research Ethics Committee reference (22/WA/0049) “Eye2Gene: accelerating the diagnosis of inherited retinal diseases” Integrated Research Application System (project ID: 242050). All research adhered to the tenets of the Declaration of Helsinki. In the United Kingdom, under the current opt-in legislation, research on retrospective deidentified data does not require reconsenting patients.

### Dataset

Our training dataset was drawn from a dataset of a total of 136 631 FAF images from 4554 IRD patients from Moorfields Eye Hospital, captured using the Heidelberg Spectralis imaging platform. All data collected for this study were 55° 486 nm blue autofluorescence images captured on Heidelberg Spectralis OCT. Standard scanner settings were used with an average of 15 captures.

**Figure 1:**
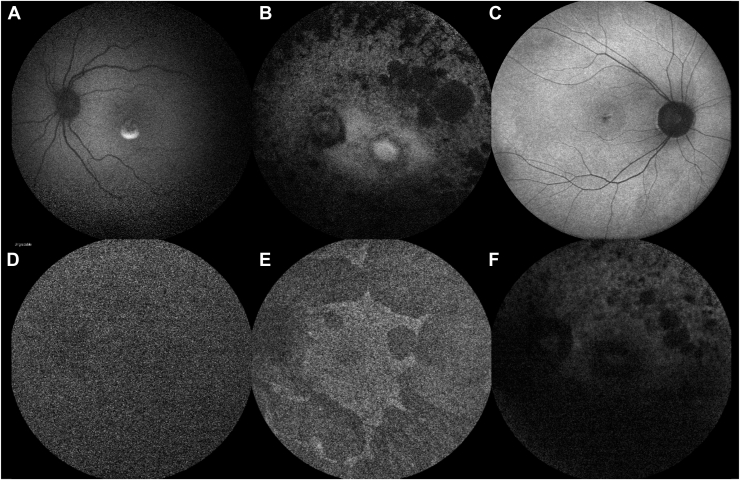
Example images annotated as (**A), (B),** and (**C)** gradable (acceptable quality), and (**D), (E),** and (**F)** ungradable (poor quality).

From this dataset, 2445 images were selected at random and then labeled by a team of 3 graders (G1, G2, and G3), with 815 images assigned to each grader. All graders were research fellows with >5 years’ experience in medical retina and had extensive experience with grading FAF scans for IRDs. Annotation was performed using 2 defined criteria for image quality, as outlined in Table 1 and illustrated in Figure 1. This annotation was done over the course of 3 weeks using an instance of the Label Studio tool,^17^ which was hosted on our online grading platform (grading.readingcentre.org).

**Table 1:** Definition of the Quality Assessment Criteria

Assessment	Criteria	Feature Visibility
Gradable (acceptable quality to a grader)	Image is sufficient to discern the relevant features with >50% certainty	Discrimination of the optic disc is clear, and vascular arcades are visible in over ¾ of their extent.
		No opacities/shadowing impairing clear visibility of critical structures like the foveal and perifoveal areas.
Ungradable (unacceptable quality to a grader)	Image is not sufficient to discern the relevant features.	One or more anatomical features impossible to discern.

An additional 133 images were selected as a held-out test set, ensuring no patient overlap with the training set, each of which was annotated by all 3 graders. This was used to measure intergrader agreement and evaluate our algorithm. In cases where not all graders agreed on the same label for a given image, the most common label (majority voting) was used for the purposes of model evaluation. This approach helped to ensure consistency and reliability in the evaluation process. This test set was 56.3% female and 43.7% male. A distribution of associated genes is given in Figure S1 (available at www.ophthalmologyscience.org), and an age distribution is given in Figure S2 (available at www.ophthalmologyscience.org).

In addition to human grading, for each image we also calculated the SNR and Blind/Referenceless Image Spatial Quality Evaluator (BRISQUE) score, which are widely used measures of signal clarity relative to noise levels and perceptual quality of images, respectively. The SNR score was calculated using the mean pixel intensity divided by the standard deviation, expressed in decibels, to quantify image quality and consistency. The BRISQUE scores were calculated for each image using the “brisque” PyPI Python package. Brisque is a reference-less quality assessment metric that estimates perceptual quality based on spatial natural scene statistics.^18^

### Model Development

For training and evaluation of our Retinograd-AI model, we divided the data into training and pretest sets in a stratified way, ensuring a similar proportion of each class in both sets by assigning patients to each split to avoid any overlap.

We employed an Inception ResNet v2 network architecture with ImageNet pretrained weights for the network (Fig S3, available at www.ophthalmologyscience.org). The model was trained using the Adam optimizer and cross-entropy loss, with class reweighting applied to account for dataset imbalance between the 2 classes. Horizontal flipping and random rotations to increase variability of data in line with standard data augmentation practices. We have trained the model for 20 epochs, taking the best-performing weights determined by the validation loss as evaluated on the pretest data. A full list of hyper-parameter settings is given in Table S1 (available at www.ophthalmologyscience.org).

## Results

The average intergrader agreement was 0.69 (substantial agreement) as measured by Cohen kappa.^19^ A full breakdown of intergrader agreement is given in Table S2 (available at www.ophthalmologyscience.org).

**Figure 2:**
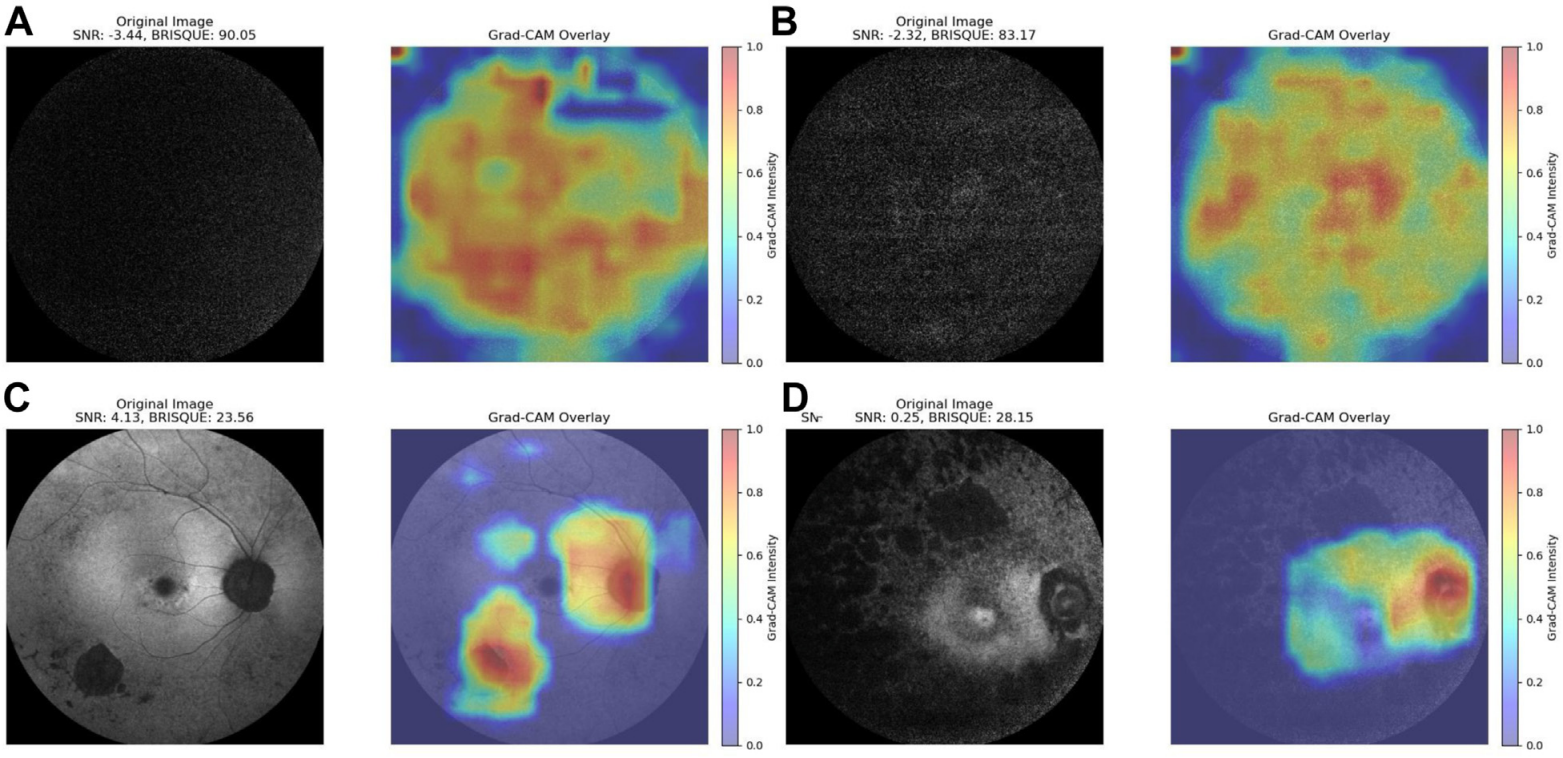
Grad-CAM plot for Retinograd-AI for selected ungradable (**A** and **B**) and gradable (**C** and **D**) images. BRISQUE = Blind/Referenceless Image Spatial Quality Evaluator; Grad-CAM = Gradient-weighted Class Activation Mapping; SNR = signal-to-noise ratio.

We evaluated Retinograd-AI on the held-out test set and compared its predictions to the grader labels, viewing the problem as a binary classification task with “gradable” being the positive class and “ungradable” being the negative class. Gradient-weighted Class Activation Mapping was applied to the trained model.^20^ The resulting Gradient-weighted Class Activation Mapping maps demonstrate that the trained model tends to focus on anatomical and pathological features in gradable images but shows diffuse focus for ungradable images (Fig 2).

**Figure 3:**
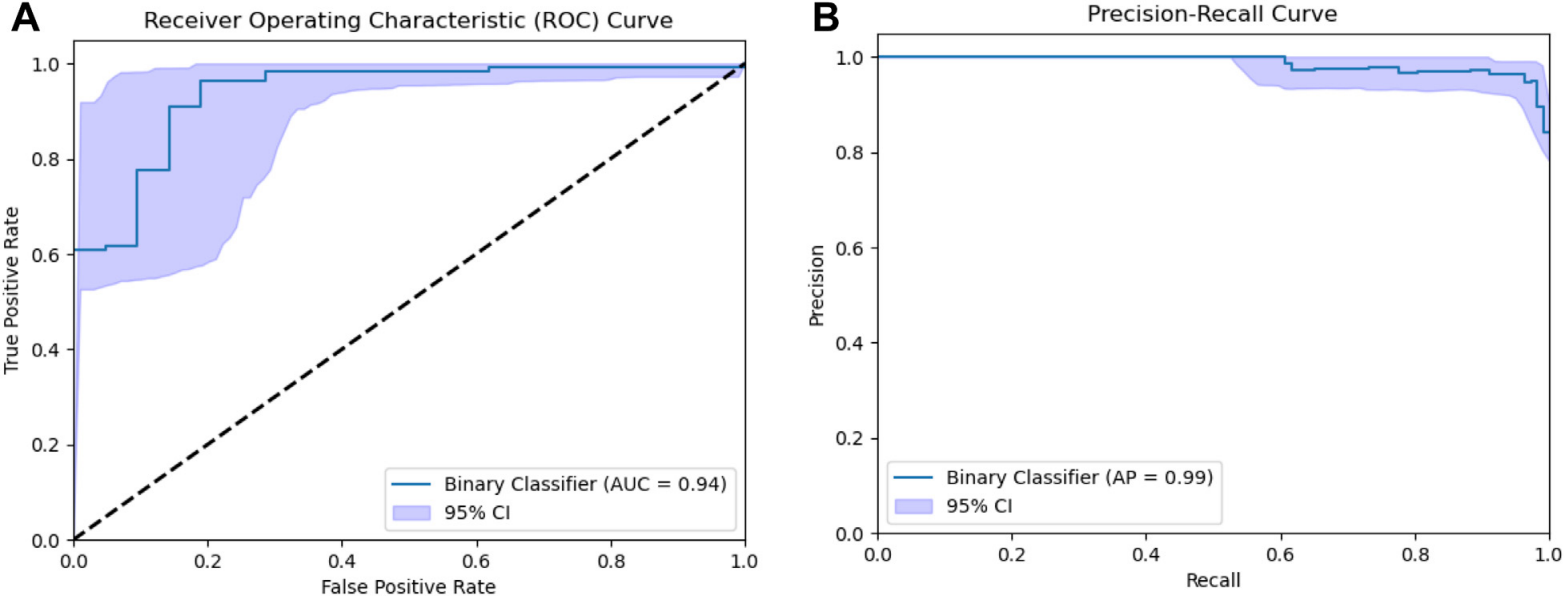
**A,** Receiver operator characteristic curve and (**B)** precision recall curve for the model predictions on the held-out test set. The shaded region denotes the 95% CI. AP = average precision; AUC = area under the curve; CI = confidence interval.

The model achieved an accuracy of 91% (CI_95_ = 85.7–95.5%) on the held-out test set, with precision of 0.96 (0.923–0.991) and recall of 0.93 (0.873–0.973). The corresponding confusion matrix is given in Table 2. The area under the receiver-operator characteristic curve was 0.94 (Fig 3). Model-grader agreement (Cohen-Kappa) was 0.69 (substantial agreement), which was comparable to the intergrader agreement. Of the cases incorrectly classified by the model as ungradable, the corresponding genetic diagnoses were *RHO* (n = 2), *RDH12* (n = 2), *MYO7A*, *PROM1*, *NDP*, and *GUCY2D*. Of the cases incorrectly classified as gradable, the corresponding genetic diagnoses were *PROM1* (n = 2), *EYS*, and *CRB1*.

**Table 2:** Model Confusion Matrix

	Model Prediction	
	Gradable	Ungradable
Grader label		
Gradable	104	8
Ungradable	4	17

Comparison of model predictions with ground-truth grader labels.

**Figure 4:**
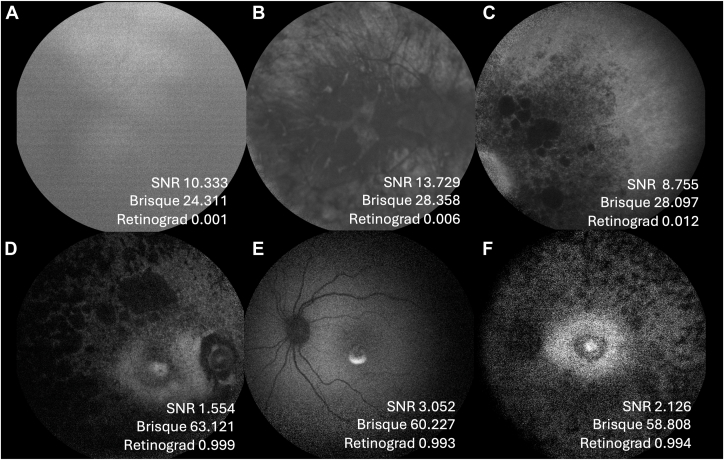
**A**, **B,** and **C** are examples of images that Retinograd-AI correctly classifies as not gradable but would be marked as gradable by SNR (>8) and Brisque (<28). Conversely, (**D)**, (**E),** and (**F)** are examples of images that Retinograd-AI correctly classifies as gradable (given that the optic disc and vascular arcades are discernible) but would be marked as ungradable by SNR (<4) and Brisque (>58). Brisque = Blind/Referenceless Image Spatial Quality Evaluator; SNR = signal-to-noise ratio.

The Cohen-Kappa coefficient compared with the grader annotations for SNR score was 0.252 (fair agreement). For the BRISQUE score, the Cohen-Kappa coefficient compared with the grader annotations was −0.181, indicative of slight agreement. A lower BRISQUE score represents better quality, hence the negative correlation. A heuristic threshold for SNR is <5, which is considered ungradable and higher would be gradable. A heurist threshold for Brisque is 50, under which an image is considered gradable and above, ungradable. As illustrated in Figure 4, the SNR and the Brisque thresholds suggest a good quality image, whereas the Retinograd-AI score correctly indicates the image is of bad quality. The converse is also illustrated in Figure 4. Overall, Retinograd-AI is more closely aligned with the human grader.

To understand how demographics might affect image quality, we applied Retinograd-AI to our full dataset of 136 631 FAF images, collected as part of the Eye2Gene study, to obtain Retinograd-AI predictions for each image. This enabled us to examine the relationship between image quality and various other data attributes such as patient age, sex, and ethnicity, and single-image classification accuracy.

**Figure 5:**
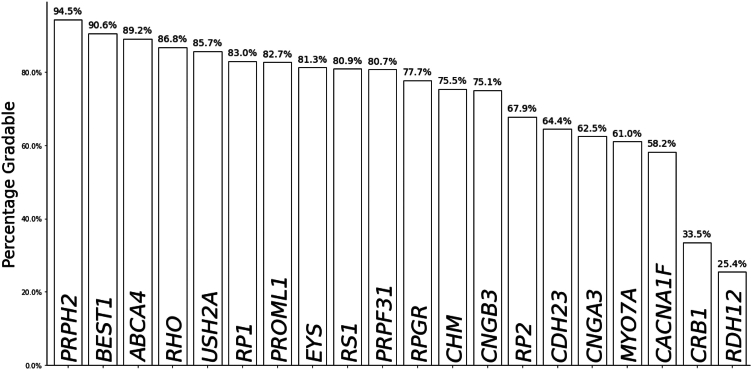
Percentage of images rated as gradable by Retinograd-AI across the 20 most common genetic diagnoses. Significant differences can be seen between certain genes, with most high- and low-gradeability genes (e.g., *PRPH2* and *ABCA4*) showing statistically significant *P* value (*P* < 0.05) above/below the mean with a chi-square test.

We observed a small but significant effect of age on Retinograd-AI-assessed image quality (ß = 0.002, *P* < 0.001), with the highest proportions of gradable images in the 30+-year-old patients and significantly higher proportions of ungradable images in younger patients, particularly in the under-15s (Table 3). There were significant differences between ethnicities in the proportion of gradable images by patient (*P* < 0.001) (Table 4). Asian and mixed patients showed the lowest proportion of gradable images (67.7%), while the highest proportion was seen in White patients (80.2%) and patients of unknown or undisclosed ethnicity (82.6%), which represented a significant fraction of cases. There was also a significant difference between male and female patients (excluding patients with X-linked genes) with images being rated as gradable 77.1% of the time for male patients and 82.3% for female patients (odds ratio = 1.43, *P* < 0.001). There was also substantial variation in image quality across different patient genotypes, with the highest proportion of gradable images found in patients with a disease-causing variant in *PRPH2*, with 93.1% of images rated as gradable, and the lowest being *RDH12,* where only 27.1% of images were rated as gradable (Fig 5).

**Table 3:** Comparison of Patient Age with Gradability

Age Range	% Gradable
0–15	39.0%
15–30	66.2%
30–50	82.5%
50–70	80.2%
70+	79.2%

**Table 4:** Comparison of Ethnicity with Mean Percentage of Gradable Images by Patient

Ethnicity	N	% Gradable
Asian or Asian British	293	67.7% (±4.1)
Black or Black British	89	74.5% (±6.9)
Mixed	30	67.8% (±14.0)
Other ethnic groups	278	78.9% (±3.7)
Unknown	240	82.6% (±3.7)
White	991	80.2% (±1.8)

**Figure 6:**
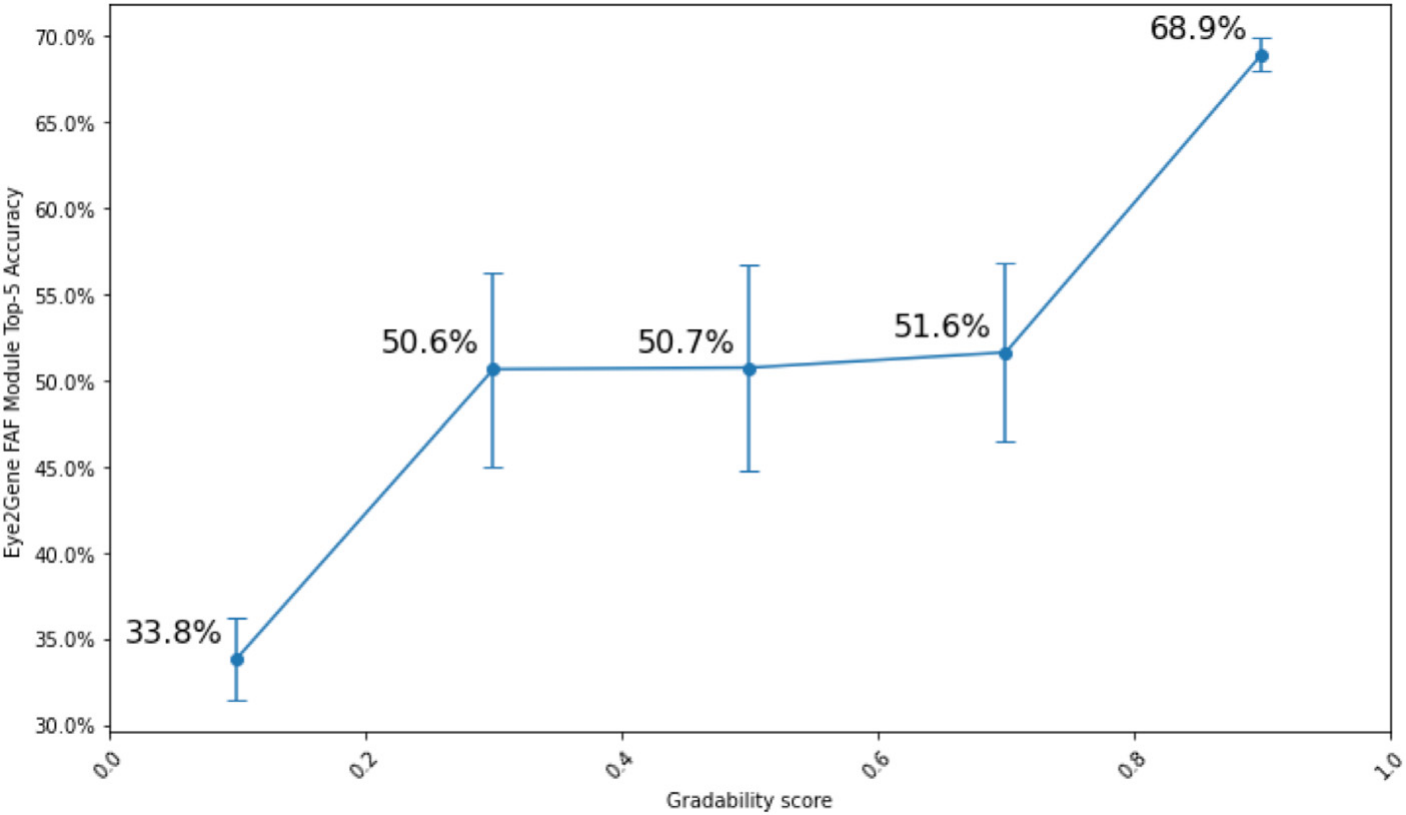
Comparison of Eye2Gene single-image FAF module top-5 gene classification accuracy compared with gradability probability (gradability score) from Retinograd-AI. All images were ranked by the probability output of Retinograd-AI and divided into 5 buckets. For each bucket, the per-bucket gene classification accuracy and standard error were calculated and plotted. FAF = fundus autofluorescence.

To assess how image gradability affects the performance of AI models, we compared the Retinograd-AI-assessed image gradability to the gene-classification accuracy of a single FAF module of Eye2Gene,^6^^,^^7^ evaluating at the image-level rather than the patient-level. We found that images classified as gradable by Retinograd-AI had a top-5 gene classification accuracy of 68.9%, while images classified as ungradable had a substantially lower accuracy of 33.8%. Figure 6 shows how gene-classification accuracy compares with the raw probability output of Retinograd-AI, showing that higher-gradability score corresponds with higher gene-classification accuracy.

## Discussion

Herein, we present Retinograd-AI, the first retinal image quality assessment model for FAF imaging and the first image quality assessment tool developed specifically for IRDs. We have open-sourced our algorithm to make it available to other researchers at https://github.com/Eye2Gene/retinograd-ai.

Retinograd-AI enabled us to automatically annotate our entire database of FAF images in IRDs from Moorfields Eye Hospital, an otherwise unfeasible task to perform manually. These data enabled us to gain valuable insights into image quality variability in relation to parameters such as patient age, sex, and genotype, which historically have been difficult to separate out due to previously unquantified influences.

As might be expected, we found that younger (0–15-year-olds) age groups had a smaller proportion of gradable images than other age groups, which could be associated with the challenges of imaging the retinas of younger individuals affected by IRDs. For IRD genotypes, we found that genotypes that are earlier onset, affecting the posterior pole, such as *RDH12,* or causing widespread degeneration, such as *CRB1,* had a lower proportion of gradable images, as did genotypes that tend to present with secondary cataract, severe phenotypes, or high myopia, such as *MYO7A*, *NR2E3,* and *CACNA1F*. Achromatopsia genotypes such as *CNGB3* and *CNGBA3* often have nystagmus and photo aversion, which could also explain a lower proportion of gradable images in those genotypes. This suggests that image gradability could be an important input into gene-classification models, although the current favored approach is to limit uncertainty by training AI on higher-quality images.^6^

Changes in the data quality between test set and routine clinical care settings could have a large impact on model performance, leading to substantially lower routine clinical care accuracy than expected, carrying implications for safety and efficacy. Automated image quality assessment tools, such as Retinograd-AI, can have an important role to play in addressing this, both by identifying variations in image quality between different settings and patient populations and by prescreening images at point of use to reject poor-quality images.

The impact of image quality on model performance also has ramifications for the reporting of performance of machine learning models on FAF imaging. The accuracy of a given model can be substantially different depending on the quality of the data in the evaluation set, meaning aggressive quality filtering can lead to artificially inflated accuracy (and other metrics), which may not be representative of the performance on routine clinical care datasets. This highlights the need for more standardized metrics of image quality and gradability to enable greater comparability across datasets.

Retinograd-AI can also be used in other scenarios where image quality is important, but expert feedback is not immediately available, for example, in collecting data for clinical trials. In these cases, Retinograd-AI could provide near real-time feedback to the operator about the quality of the captured images and whether it is sufficient for downstream analysis or whether repeat imaging is recommended.

While Retinograd-AI demonstrated promising performance, there are still a number of limitations that warrant further consideration. Firstly, FAF imaging is used across multiple conditions; however, Retinograd-AI was evaluated only on data from IRD patients, meaning applicability to other conditions has not yet been assessed. Additionally, the use of retrospective data meant we were unable to experiment with image acquisition protocols for the purpose of this study. It would be interesting to assess how scanner parameters affect imaging quality. Finally, Retinograd-AI also cannot currently explain why the images are of poor quality, which would be useful for identifying systematic issues and informing improvements in imaging protocols, as well as providing enhanced operator feedback.

Furthermore, as we have demonstrated, filtering for quality can risk disproportionately filtering out patients with certain genes, age groups, sexes, and ethnicities. Additionally, even objectively poor-quality imaging may still be useful. For example, clinicians may identify diagnostic features that are still interpretable despite imperfections and use contextual knowledge to compensate for deficiencies. Hence, care should be taken by practitioners when applying Retinograd-AI and other quality filtering approaches to consider if it is appropriate for their use case.

Given the diversity in age and phenotypes of IRDs, Retinograd-AI is a robust starting point for building gradability models for FAF imaging for other conditions where FAF is commonly used, such as geographic atrophy and central serous chorioretinopathy, potentially via transfer learning using Retinograd-AI weights as a starting point.

We expect automatic gradability annotations to prove invaluable to future image classification and segmentation tasks, as imaging quality is a significant confounder for many image-derived metrics.

In the future, we aim to improve Retinograd-AI by incorporating additional data from other conditions, as well as extend our approach to further imaging modalities.

## Code Availability

The source code for the Retinograd-AI model architecture training and inference is available from https://github.com/Eye2Gene/retinograd-ai.

## Data Availability

The data that support the findings of this study are divided into 2 groups: published data and restricted data. Published data are available from the GitHub repository. Restricted data are curated under a UCL Business-owned license and cannot be published to protect patient privacy and intellectual property. Synthetic data derived from the test data has been made available on Figshare^21^ and at https://github.com/Eye2Gene/retinograd-ai.
