# Supplementary material for: Retinograd-AI: An Open-Source Automated Fundus Autofluorescence Retinal Image Gradability Assessment for Inherited Retinal Diseases
Source: Ophthalmol Sci. 2025 Jun 4;5(6):100845. doi: 10.1016/j.xops.2025.100845 (PMC12309597; doi:10.1016/j.xops.2025.100845)
Supplement: Figure S1 [file mmc1.docx]

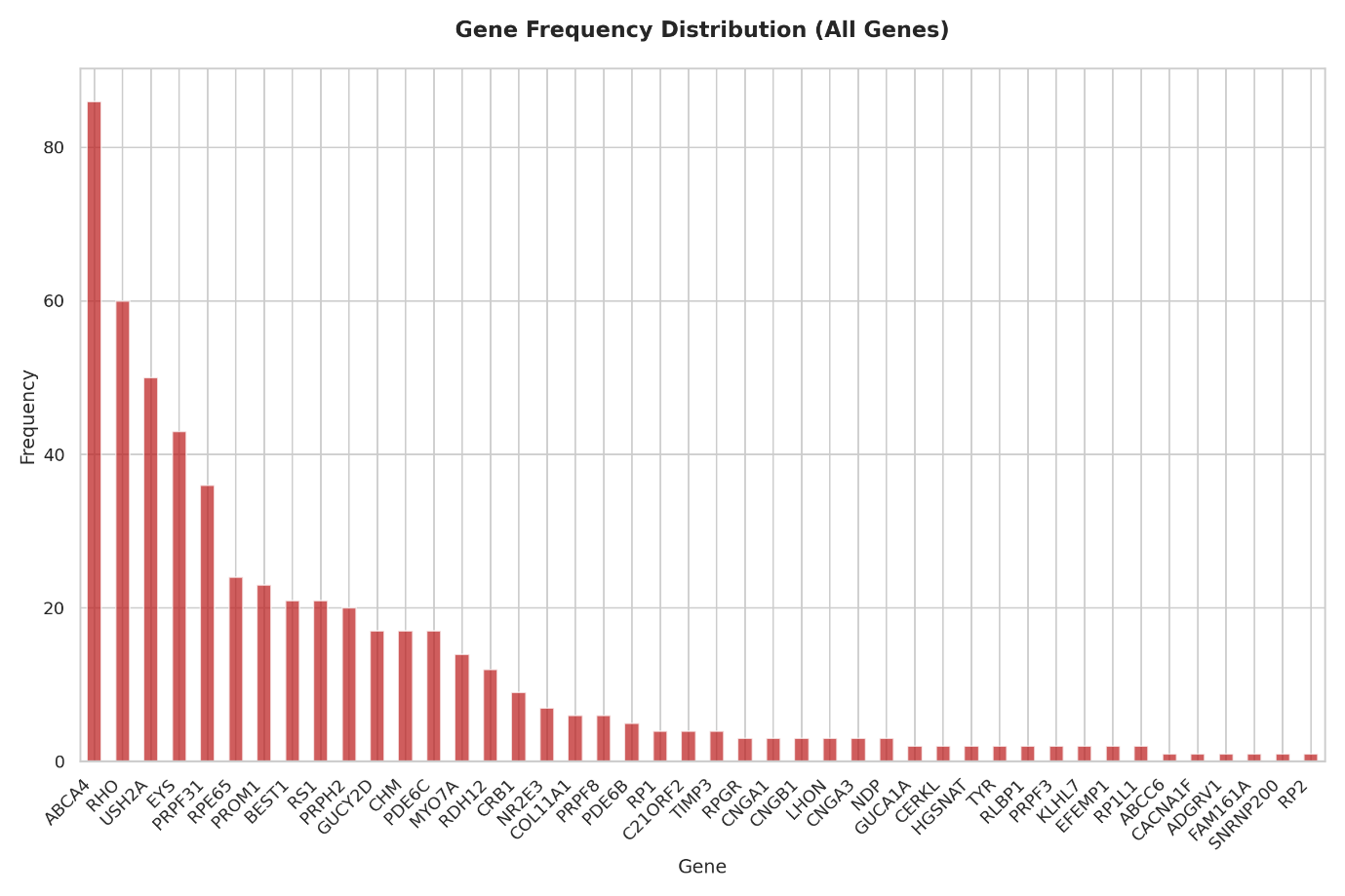


**Supplementary Figure 1**: Gene distribution across patients whose images were included in the held-out test set for Retinograd-AI.
