# Supplementary figures and images for: Retinograd-AI: An Open-Source Automated Fundus Autofluorescence Retinal Image Gradability Assessment for Inherited Retinal Diseases
Source: Ophthalmol Sci. 2025 Jun 4;5(6):100845. doi: 10.1016/j.xops.2025.100845 (PMC12309597; doi:10.1016/j.xops.2025.100845)

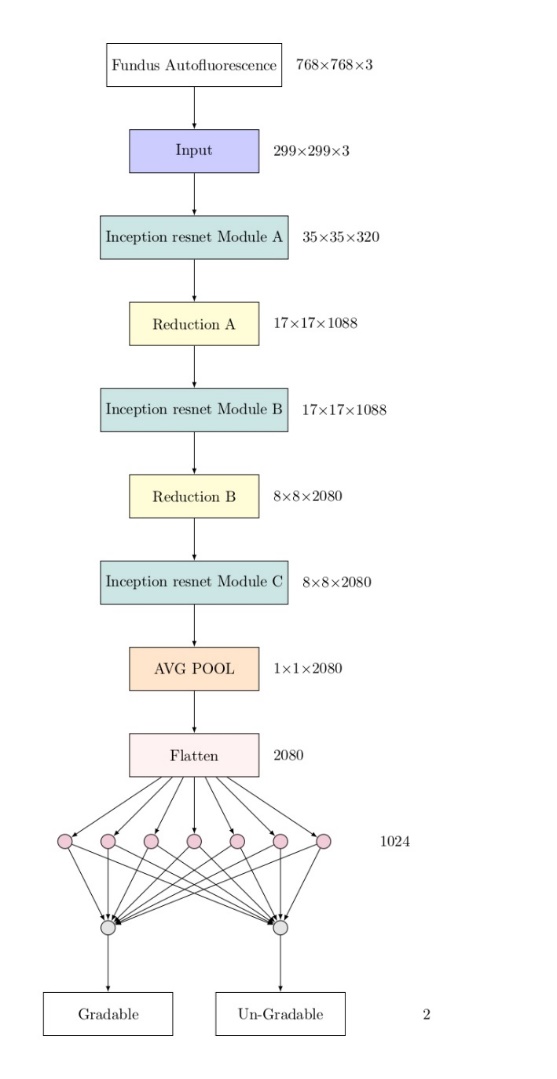


**Supplementary Figure 3:** Inception Resnet V2 architecture diagram for Retinograd-AI.

Supplement: Figure S3 [file mmc3.docx]
