# Supplementary material for: Retinograd-AI: An Open-Source Automated Fundus Autofluorescence Retinal Image Gradability Assessment for Inherited Retinal Diseases
Source: Ophthalmol Sci. 2025 Jun 4;5(6):100845. doi: 10.1016/j.xops.2025.100845 (PMC12309597; doi:10.1016/j.xops.2025.100845)
Supplement: Table S1 [file mmc4.docx]

**Supplementary Table 1**: List of hyperparameter settings used for training the Retinograd-AI neural network.

| **Parameter** | **Value** |
| --- | --- |
| Architecture | inception_resnet_v2 |
| Batch size | 4 |
| Image size | (768,768) |
| Train Epochs | 20 (Early stopping as no validation loss improvement) |
| Optimiser | Adam |
| Loss | Weighted categorical cross-entropy |
| Learning rate | 1e-5 |
| Augmentations | Horizontal flipping, Random rotations |
| Pretrained weights | ImageNet |
| Class weights for weighted loss | [0:3.7716, 1:0.5764] |
| Model saving criteria | Validation loss |
