# Supplementary material for: Retinograd-AI: An Open-Source Automated Fundus Autofluorescence Retinal Image Gradability Assessment for Inherited Retinal Diseases
Source: Ophthalmol Sci. 2025 Jun 4;5(6):100845. doi: 10.1016/j.xops.2025.100845 (PMC12309597; doi:10.1016/j.xops.2025.100845)
Supplement: Table S2 [file mmc5.docx]

**Supplementary Table 2**: Inter-grader confusion matrix G=Gradable, U=Ungradable

| **Grader 1 vs Grader 2** | | **Grader 2** | | **Grader 1 vs Grader 3** | | **Grader 3** | | **Grader 2 vs Grader 3** | | **Grader 3** | |
| --- | --- | --- | --- | --- | --- | --- | --- | --- | --- | --- | --- |
|  |  | **G** | **U** |  |  | **G** | **U** |  |  | **G** | **U** |
| **Grader 1** | **G** | 110 | 7 | **Grader 1** | **G** | 113 | 4 | **Grader 2** | **G** | 110 | 5 |
|  | **U** | 5 | 11 |  | **U** | 2 | 14 |  | **U** | 5 | 13 |
